# Upcycling agricultural residues into polyhydroxyalkanoates through pilot-scale mixed microbial culture processes

**DOI:** 10.3389/fbioe.2026.1952375

**Published:** 2026-10-05

**Authors:** Giovanna Pesante, Elvis Montagnese, Claudia Magonara, Alessia Zucca, Gaia Salvatori, Lionel Nguemna Tayou, Luca Di Leva, Matteo Migliorini, Marianna Villano, David Bolzonella

**Affiliations:** 1 Department of Biotechnology, University of Verona, Verona, Italy; 2 Department of Chemistry, Sapienza University of Rome, Roma, Italy

**Keywords:** agricultural residue valorisation, downstream processing, enzymatic purification, mixed microbial cultures, pilot-scale biorefinery, poly(hydroxybutyrate-hydroxyvalerate) (PHBV), polyhydroxyalkanoates (PHAs)

## Abstract

Polyhydroxyalkanoates (PHAs) produced by mixed microbial cultures (MMCs) represent a promising alternative to conventional plastics, enabling the valorisation of agricultural residues within circular biorefineries. However, despite extensive laboratory-scale research, integrated pilot-scale studies encompassing the complete production chain, from feedstock fermentation to downstream polymer recovery, remain scarce. In this study, poly (hydroxybutyrate-hydroxyvalerate) (PHBV) production from agricultural residues was investigated in a pilot plant integrated within a full-scale agricultural anaerobic digestion facility. The process included acidogenic fermentation, microbial selection, PHBV accumulation, biomass recovery, while downstream extraction and purification were evaluated at laboratory scale on pilot-produced biomass. A total of 15 pilot-scale campaigns were carried out, allowing progressive optimisation. Two representative campaigns were selected: Batch 12, fed with a synthetic carboxylic acid (CA) mixture, and Batch 14, which used a real agricultural feedstock derived from cattle slurry and maize silage. Acidogenic fermentation of the agricultural substrate produced up to 16.7 gCOD/L of CAs, with valeric acid accumulation, providing suitable precursors for PHBV synthesis. Microbial community analysis confirmed the successful enrichment of recognised PHA-storing genera, including *Corynebacterium*, *Lampropedia*, *Paracoccus* and *Thauera*. Pilot-scale accumulation produced PHBV with a 3-hydroxyvalerate (HV) content of 14%–26% (w/w), while intracellular PHA contents reached 26% and 30% (w/w) for Batch 12 and Batch 14, respectively. More than 1 kg of PHBV-rich biomass was recovered in both campaigns, while 10.2 kg throughout the 15 campaigns. Among the downstream treatments investigated, a NaOH-HAc protocol provided the best compromise between polymer purity, molecular weight preservation, operational simplicity and process economics. For the synthetic-feedstock biomass, PHA content increased from 26% to a purity of 58% after NaOH-HAc treatment, whereas purification of the real-feedstock biomass reached 37%, highlighting the greater complexity of processing heterogeneous waste-derived substrates. Although polymer purities remained lower than those typically achieved with pure cultures, the PHBV obtained exhibited HV contents that may favour flexibility and support its potential use in agricultural applications requiring flexible compounds. Overall, this work provides one of the few integrated pilot-scale demonstrations of MMC-based PHBV production from agricultural residues, identifies downstream processing as a key bottleneck and contributes to advancing the industrial implementation of sustainable waste-based PHA biorefineries.

## 1 Introduction

The increasing environmental concerns associated with fossil-based plastics, together with the growing demand for eco-sustainable materials, have intensified research efforts towards the development of biodegradable and bio-based polymers. Among these, polyhydroxyalkanoates (PHAs) have emerged as one of the most promising alternatives to conventional petrochemical plastics due to their biodegradability, biocompatibility, and thermoplastic properties (Kourmentza et al., 2017; Chen and Patel, 2012). PHAs are intracellular polyesters naturally synthesised by numerous microorganisms as carbon and energy storage compounds under conditions of nutrient limitation and carbon excess. Depending on their monomeric composition, PHAs can display a broad range of mechanical and thermal properties suitable for applications in packaging, agriculture, biomedicine, and consumer products (Muhammadi et al., 2015).

Among the different PHA types, poly(3-hydroxybutyrate-co-3-hydroxyvalerate) (PHBV) has received considerable attention due to its improved flexibility and reduced brittleness compared with polyhydroxybutyrate (PHB). Incorporating 3-hydroxyvalerate (3HV) units into the polymer chain enhances ductility and processability, thereby expanding the range of potential industrial applications (Laycock et al., 2013; Volova et al., 2023). However, despite their attractive properties, the large-scale commercialization of PHAs remains constrained by high production costs, which are still significantly higher than those of conventional plastics. Feedstock costs and downstream processing are among the major contributors to the overall economic burden of PHA production processes (Choi and Lee, 1999; Koller et al., 2008).

To overcome these limitations, increasing attention has been devoted to the valorisation of agricultural and food-processing by-products (agri-food residues), as well as other organic waste streams, as low-cost substrates for PHA synthesis. In this context, mixed microbial cultures (MMC) represent a particularly attractive platform compared with pure microbial cultures because they do not require sterile operating conditions and can efficiently convert heterogeneous waste-derived substrates into PHAs (Serafim et al., 2008). MMC-based PHA production generally relies on a three-stage process consisting of acidogenic fermentation for carboxylic acid (CA) production, enrichment of PHA-storing microorganisms through feast–famine selection strategies, and a final accumulation phase under nutrient-limited conditions (Reis et al., 2003). This approach enables the integration of PHA production within existing waste management infrastructures, thereby contributing to circular bioeconomy strategies.

Several studies have demonstrated the feasibility of producing PHAs from agri-food residues, wastewater sludge, and lignocellulosic by-products using MMC (Bengtsson et al., 2008; Villano et al., 2010; Valentino et al., 2017; Filippou et al., 2025; Rodrigues et al., 2024). In recent years, agricultural residues have attracted particular interest due to their widespread availability and high organic content. Their conversion into CAs via anaerobic fermentation provides an efficient route to generate suitable carbon-rich substrates for MMC-based PHA production while simultaneously reducing waste disposal burdens and greenhouse gas emissions (Vázquez-Fernández et al., 2022, Atasoy et al., 2018). Within this framework, the Horizon Europe project AgriLoop aims to promote the upcycling of agri-food residues into high-value biobased products, including PHBV (Gontard et al., 2026).

Although numerous laboratory-scale studies have reported promising PHA yields using MMCs fed with waste-derived CAs, the transition from laboratory to pilot and industrial scale remains a critical bottleneck for technology implementation. Scale-up challenges include maintaining process stability under non-sterile conditions, handling highly heterogeneous substrates, ensuring efficient solid–liquid separation, and integrating upstream and downstream processing units into continuous operational schemes (Morgan-Sagastume et al., 2015; Valentino et al., 2019). Most MMC-PHA studies reported in the literature have been conducted at laboratory scale, typically involving reactor volumes below 100 L. Conversely, studies investigating pilot-scale MMC-based PHA production remain relatively limited and are often based on reactor volumes of only a few hundred litres.

A few pilot-scale demonstrations have highlighted the technical feasibility of integrating PHA production into wastewater treatment and biorefinery platforms. Morgan-Sagastume et al. (2015) operated one of the first pilot-scale MMC-PHA systems coupled with municipal wastewater treatment, demonstrating the potential to enrich PHA-storing biomass under dynamic feeding conditions. Similarly, Valentino et al. (2019) evaluated pilot-scale PHA production from fermented organic waste streams and emphasized the importance of operational control during the selection and accumulation phases. More recently, efforts have focused on integrating PHA production into existing anaerobic digestion infrastructures to exploit available fermentation units and waste-handling systems (Valentino et al., 2019; Papa et al., 2020). Nevertheless, the number of studies investigating pilot-scale production of PHBV from agricultural residues using MMC remains scarce, particularly across the entire process chain from feedstock fermentation to biomass recovery, extraction and purification, since most investigations have focused only on individual process steps. Consequently, the scalability of the overall process and the interaction between upstream and downstream operations are still poorly understood.

Besides fermentation performance, downstream processing remains a major technological and economic bottleneck hindering PHA commercialization (Pagliano et al., 2021). Conventional extraction methods are commonly based on halogenated solvents such as chloroform or on strong oxidizing chemicals, raising concerns about environmental sustainability, polymer degradation, solvent recovery, and process safety (Jacquel et al., 2008). In MMC-derived biomass, downstream processing is further complicated by the heterogeneous microbial composition and the presence of large amounts of non-PHA cellular material (NPCM), extracellular polymeric substances, and inorganic residues. Therefore, the development of efficient and scalable purification strategies compatible with MMC-based processes is essential for improving overall process sustainability. In recent years, increasing attention has been devoted to greener extraction approaches based on chemical digestion, mechanical disruption, or enzymatic treatments (Samorì et al., 2015; Mannina et al., 2020). Enzymatic treatments are particularly attractive because they operate under milder conditions and may selectively hydrolyse NPCM while preserving polymer molecular weight and material properties. Proteases such as alcalase have been investigated for the degradation of proteinaceous biomass fractions, while lysozyme and phospholipases can contribute to the disruption of bacterial cell walls and membranes (Mannina et al., 2020; Kapritchkoff et al., 2006). However, to the best of our knowledge, studies investigating enzymatic extraction strategies for PHAs produced by MMCs from agri-food residues or other organic waste-derived substrates are still lacking.

The present work builds upon a recently published laboratory-scale study focused on PHBV production from agricultural residues using MMC and CA-rich substrates generated through acidogenic fermentation. Rather than focusing on a single process step, this study evaluates the scalability of the entire production chain under pilot-scale conditions. The process was implemented in a cubic-metre-scale pilot plant integrated within a real agricultural cooperative hosting a full-scale anaerobic digestion facility. The work investigated the entire production chain, including CA generation from agricultural residues, MMC selection and PHBV accumulation in pilot-scale reactors, and biomass recovery, while downstream extraction and purification strategies were subsequently evaluated at laboratory scale on biomass produced in the pilot plant. Particular attention was devoted to the evaluation of downstream processing strategies for MMC-derived biomass produced at pilot scale. Different chemical, enzymatic, and combined chemical–enzymatic treatments were investigated at laboratory scale to assess their efficiency in improving polymer purity while preserving PHBV composition and quality. The study therefore provides one of the few comprehensive assessments of pilot-scale MMC-based PHBV production from agricultural residues coupled with laboratory-scale evaluation of downstream extraction and purification strategies applicable to MMC-derived biomass. Overall, the work contributes to bridging the gap between laboratory research and industrial implementation of MMC-based PHA biorefineries within the framework of circular bioeconomy and agricultural waste valorisation.

## 2 Materials and Methods

### 2.1 Experimental setup

The experimental activity was conducted in a pilot plant for PHA production, installed within the agricultural cooperative La Torre in Isola della Scala, Verona, Italy. The cooperative hosts two full-scale anaerobic digestion plants, each 1 MW in size, that treat around 150 tons per day of agricultural waste. The pilot plant was equipped for the integrated production of CAs and PHAs and was continuously controlled by a programmable logic controller, allowing partially remote operation and process monitoring. The plant comprised the following units (Figure 1):

1. External storage tank for feedstock preparation prior to fermentation (volume of 2 m^3^).
2. Fermentation unit for the conversion of agricultural residues into a CA-rich stream for PHA production (volume of 5 m^3^).
3. Screw press for solid-liquid separation of the obtained CA-rich stream, followed by manual clarification through a fine-mesh mesh.
4. Carbon storage unit for the clarified CA-rich stream (volume of 2 m^3^).
5. Sequential batch reactor (SBR) units, consisting of SBR1 and SBR2. SBR1 (volume of 2 m^3^) was used for the selection of PHA-storing biomass, whereas SBR2 (volume of 1.2 m^3^) was utilised for the PHA accumulation phase.
6. Biomass recovery unit, used to recover and concentrate the biomass by flocculation and decantation of the liquid (volume of 1.5 m^3^).

**FIGURE 1:**
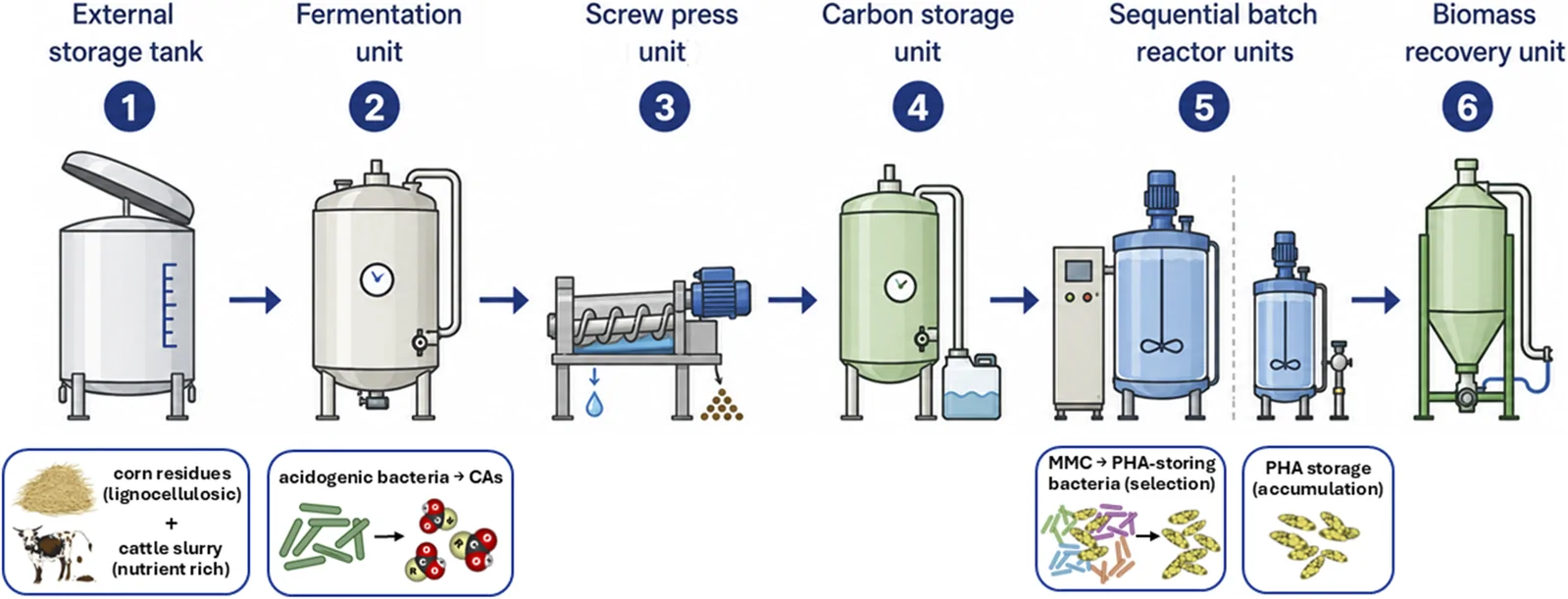
Schematic representation of the pilot plant developed for PHBV production from agricultural residues.

After recovery, metabolic activity was quenched by acidification with sulfuric acid up to pH 2, and the biomass was transferred to the University of Verona laboratories for PHA extraction and purification.

The experimental campaign comprised 15 pilot-scale campaigns (named batches), covering the entire process chain, from feedstock preparation to biomass selection, PHA accumulation, biomass recovery, and downstream extraction and purification. The first campaigns were mainly devoted to process optimisation and validation of the operating procedures, whereas the subsequent campaigns were performed under progressively stabilised operating conditions. For the purposes of this manuscript, only two representative pilot-scale campaigns (Batch 12 and Batch 14) are described in detail, as they represent the two operating scenarios investigated during the project, namely, the use of synthetic (Batch 12) and real agricultural feedstocks (Batch 14). Results from all pilot-scale campaigns are briefly presented in the Results and Discussion section, while the detailed discussion focuses on the two selected batches.

### 2.2 Feedstock preparation and acidogenic fermentation

The substrate used for PHA production during Batch 12 was a synthetic mixture (9.7 gCOD/L) of CAs (acetic 75.0%, propionic 13.0% and butyric acid 12.0%) calibrated to obtain the targeted content of HV (20%–30%) in the polymer (Magonara et al., 2025). The C:N:P ratio of this feed, expressed as gCA_COD_, was 100:10:1, obtained by adding nitrogen and phosphorus in the form of ammonium bicarbonate and phosphoric acid. The synthetic feed was directly prepared in the carbon storage unit.

In Batch 14, the system switched to a real feedstock originating from agricultural residues produced by the agricultural cooperative. These consisted of a mixture of cattle slurry and corn residues (maize silage obtained by anaerobic fermentation of chopped corn biomass, compacted and stored under oxygen-free conditions) mixed at a 50:50 ratio on a total volatile solids (TVS) basis, to which water was added in order to reach 8% of total solids (TS), thereby reducing viscosity and preventing pipe clogging.

The residues were placed in the external storage tank, which had a maximum operating volume of 2.0 m^3^ and was equipped with an adjustable stirrer, an internal solution recirculation system, and a weighing system to ensure accurate monitoring and control of the feedstock supplied to the process. Substrate homogenization was achieved using a mechanical mixer (diameter 1.3 m, height 1.8 m, rotational speed 40 rpm). A centrifugal grinder pump (Vidotto Dissipatori, model TM451) was used to homogenise and transfer the feedstock to the fermenter through an 80 mm diameter pipeline.

The fermentation unit was made of stainless steel, with a total volume of 5 m^3^ and a working volume of 1.7 m^3^, and was operated in semi-continuous mode. The reactor was fed daily with 0.59 m^3^ of mixed substrate from the external storage tank. Temperature was controlled by three electric heating elements (3 kW each) and a hot-water jacket, coupled with a PT100 temperature probe (±0.1 °C). Mesophilic conditions (35 °C) were maintained throughout the fermentation process, which operated at an average organic loading rate (OLR) of 15 kgCOD·m^-3^·day^-1^and a hydraulic retention time (HRT) of 2.8 d. These operating conditions were selected to maximise the conversion of particulate COD into CAs, thereby increasing carbon availability for the subsequent PHA production process. Clarified digestate from the full-scale anaerobic digestion plant (TS 6.3% ± 0.1%, TVS 3.7% ± 0.2%) was used as inoculum.

Following fermentation, the anaerobic fermentate underwent solid-liquid separation using a screw press (WAMGROUP, model SEPCOM SEPBIOH11502) with a nominal treatment capacity of 5 m^3^ h^−1^. The resulting liquid fraction (approximately 0.4 m^3^ d^−1^) was subsequently filtered through a 0.22-mm mesh only in Batch 14 to remove residual suspended solids. Membrane filtration was intentionally avoided because it was considered impractical for the treatment volumes required at pilot scale. The clarified liquid was then transferred to the carbon storage unit, which had a maximum capacity of 2 m^3^, was equipped with mechanical agitation and operated at ambient temperature (25 °C). This unit was used to store either the clarified CA-rich stream or the synthetic carbon source prior to PHA production.

### 2.3 Selection of PHA-storing biomass

SBR1 was employed for the biomass selection phase aimed at enriching a microbial consortium capable of storing PHAs. The reactor had a total capacity of 2 m^3^, was equipped with a blower for air diffusion, and was operated at a constant temperature of 25 °C. The inoculum consisted of activated sludge collected from a full-scale wastewater treatment plant in Verona, Italy, which was enriched under aerobic dynamic feeding conditions to select PHA-storing microorganisms (Pesante et al., 2026). The cultivation process was conducted under aerobic conditions with different operative parameters in the different batches. In particular, in batch 12, when synthetic CAs were used as feeding, the target hydraulic retention time (HRT) was 3 days. The operational strategy consisted of four feast–famine cycles per day, each lasting 6 h, with a feast-to-famine ratio (F/F) of 0.2. The organic loading rate (OLR) was initially set at 3.1 kgCOD·m^−3^·day^−1^. When operating with real substrates, like in batch 14, 2 cycles per day were carried out for a corresponding HRT of 6 days, while the feast-to-famine ratio decreased to 0.1 and the organic loading was set to 1.7 kgCOD·m^−3^·day^−1^. Aeration was provided by linear membrane blowers, which maintained dissolved oxygen concentrations between 8 and 10 mg O_2_ L^−1^ while ensuring complete mixing. Reactor operation, including temperature (25 °C), pH (8.5–9.0) and feeding cycles, was fully controlled by the programmable logic controller (PLC). Temperature and pH were controlled at 25 °C and between 8.5 and 9.0, respectively. The synthetic feedstock used for SBR1 (but not for SBR2) in Batch 12 was enriched with ammonium bicarbonate and phosphoric acid to achieve a COD:N:P ratio of 100:10:1, thereby ensuring the availability of nitrogen and phosphorus required for microbial metabolic growth during the selection process. This supplementation was unnecessary for Batch 14, as the real feedstock naturally provided sufficient nutrient concentrations. At the end of the feast phase, the selected biomass was withdrawn and transferred to SBR2 for the subsequent accumulation step.

The feast-to-famine ratio (F/F) was determined according to the following equation:

$$\frac{F}{F}\left(\min/\min\right)=\frac{Feast\;phase\;lenght}{Famine\;phase\;lenght}$$

### 2.4 PHA accumulation and biomass recovery

The SBR2 was used for the PHA synthesis and accumulation phase. The reactor had a working volume of 1.2 m^3^, was equipped with a blower for air diffusion, and was maintained at a constant temperature of 25 °C. PHA accumulation by the selected microbial consortium was carried out in fed-batch mode using a multi-spike feeding strategy to maintain a CA concentration of 0.5 g CA_COD_ L^−1^ in the bioreactor bulk throughout the accumulation phase. The termination of each feeding phase was determined by monitoring the dissolved oxygen (DO) profile. A decrease in DO during substrate feeding indicated active substrate uptake, whereas the subsequent increase in DO indicated substrate depletion and the transition from the feast to the famine phase. Thus, the end of the feast phase was identified based on the recovery of DO, which was used as an operational indicator of substrate depletion (Wang et al., 2017). The initial working volume in SBR2 was set at 1.0 m^3^, and operational conditions (temperature, pH, and DO) were maintained consistent with those adopted in SBR1.

Following operation of SBR2, the biomass was conveyed to a dedicated biomass recovery unit for concentration prior to downstream processing. This unit, with a capacity of 1.5 m^3^, was equipped with a mechanical stirrer and constructed from chemically resistant plastic materials. Biomass concentration was achieved by flocculation using a polyelectrolyte (SCAEFLOC CL 1908) at a dosage of 0.23–0.29 g L^−1^, followed by liquid–solid separation through gravity settling and solid retention using a 1 mm mesh sieve.

The recovered biomass subsequently underwent a quenching step by the addition of 95% H_2_SO_4_ until the pH reached a value of 2, thereby arresting microbial metabolic activity. Finally, the treated biomass was transferred to the laboratories at the University of Verona for downstream purification.

### 2.5 PHA extraction and purification

Preliminary extraction and purification trials were first carried out at laboratory scale using PHA-rich biomass produced in the pilot plant, to identify the most effective downstream processing strategy prior to pilot-scale implementation. Three categories of treatments were investigated: (i) chemical treatments based on NaOH, acetic acid, and hydrogen peroxide; (ii) enzymatic treatments employing alcalase, lysozyme, and phospholipase A2; and (iii) combined chemical–enzymatic treatments in which enzymatic protocols were applied following NaOH treatment. The efficiency of each protocol was evaluated in terms of polymer purity and HV content.

Based on the laboratory-scale screening, the combination of NaOH and acetic acid was selected for pilot-scale implementation during the two accumulation batches investigated in this study. Although NaOH–alcalase provided the highest PHA purity, the NaOH–HAc protocol was selected for pilot-scale application based on its combination of purification performance, polymer preservation, operational simplicity and the lower material requirements associated with chemical treatment.

Prior to extraction and purification treatments, the selected biomass samples underwent a preliminary washing step in order to remove soluble salts and residual impurities potentially interfering with downstream processing. Biomass samples were washed twice with distilled water by centrifugation (6900 rpm, 10 min, 4 °C; Eppendorf 5810R). The recovered wet pellet was subsequently subjected to the protocols described below.

#### 2.5.1 Chemical treatments

The alkaline protocol was adapted from Rodrigues et al. (2022). The biomass was treated for the digestion of the NPCM with NaOH at a final concentration of 0.3 M and a biomass concentration of 20 gTS/L. The treatment was performed under controlled laboratory conditions for 4 h. After treatment, the biomass was immediately washed twice with distilled water by centrifugation (6900 rpm, 10 min, 4 °C) to remove residual alkali and solubilized non-PHA cellular material.

For acetic acid treatment, freeze-dried biomass was resuspended in distilled water to obtain a final TS concentration of 5% (w/v). Acetic acid (98%, v/v) was added at a dosage of 0.4 mL/gTS, corresponding to a final concentration of 2.0% (v/v), and the suspension was incubated overnight under agitation at 140 rpm. The treated biomass was subsequently washed with distilled water, and the recovered pellet was freeze-dried.

In selected laboratory-scale experiments, acetic acid treatment was followed by oxidation with hydrogen peroxide. After the acetic acid step, the biomass was washed twice with distilled water (6900 rpm, 10 min, 4 °C) to remove residual acid. Hydrogen peroxide was then added at a concentration of 1.5%, using a dosage ratio of 3 mL/g wet biomass, and incubated for 4 h under agitation at 140 rpm (Salvatori et al., 2023). At the end of the treatment, biomass was washed twice again with distilled water, centrifuged, and freeze-dried before characterization.

#### 2.5.2 Enzymatic and combined chemical–enzymatic treatments

Enzymatic treatments were investigated as an alternative downstream strategy to conventional chemical treatments. Three enzymes were evaluated individually and sequentially in combined protocols: alcalase (Alcalase 2.4 L, Novozymes), lysozyme (62971-10G-F, Merck), and phospholipase A2 (PLA2-001, Creative Enzymes) (Table 1). Prior to enzymatic treatment, the freeze-dried biomass underwent the same washing pretreatment described above.

**TABLE 1:** Details on the enzymes used for the enzymatic and combined chemical-enzymatic treatments.

Name	Organism of production	Activity	pH range	Temperature range	EC number
Alcalase	*Bacillus licheniformis*	2,4 AU-A/g	6.5–10.0	60 °C–75 °C	3.4.21.62
Lysozyme	*Gallus gallus* (egg white)	∼70000 U/mg	6.0–9.0	25 °C–60 °C	3.2.1.17
Phospholipase A2	*Sus scrofra* (pancreras)	10000 U/g	4.0–6.0	45 °C–55 °C	3.1.1.4

Alcalase, a serine endopeptidase, was employed to hydrolyse proteinaceous cellular material and facilitate the release of PHA granules. The enzyme was added at a final concentration of 12.5% (w/w relative to biomass) in 0.4 M phosphate buffer at pH 7.5. Biomass concentration was maintained at 5% (w/v), and hydrolysis was carried out for 240 min at 50 °C under agitation at 180 rpm (Innova 42 shaker, New Brunswick).

Lysozyme, a glycoside hydrolase, was used to hydrolyse β-(1→4) glycosidic bonds between *N*-acetylmuramic acid and *N*-acetylglucosamine residues in bacterial peptidoglycan, thereby disrupting the cell wall structure. The enzyme was applied at 5% (w/w relative to biomass) in 50 mM TRIS-HCl buffer at pH 8. The reaction was performed for 240 min at 37 °C under agitation at 180 rpm.

Phospholipase A2 (PLA2) was selected due to its ability to hydrolyse membrane phospholipids, including those associated with Gram-negative bacteria commonly present in MMCs. PLA2 was added at a dosage of 0.2 mg/g biomass in 10 mM TRIS-HCl buffer at pH 5.5. The reaction was conducted for 240 min at 50 °C under agitation at 180 rpm.

Combined chemical–enzymatic protocols were also evaluated. In the first combined protocol, lysozyme and alcalase were sequentially applied following NaOH treatment in 0.4 M phosphate buffer at pH 7.5. Lysozyme treatment was first conducted for 120 min at 37 °C and 180 rpm, followed by alcalase treatment for an additional 120 min at 50 °C and 180 rpm. In the second combined protocol, lysozyme, alcalase, and PLA2 were sequentially applied following NaOH treatment in 0.4 M BPS buffer at pH 6. Lysozyme treatment was first carried out for 120 min at 37 °C, followed by simultaneous addition of alcalase and PLA2 for the remaining 120 min at 50 °C. Enzyme concentrations were maintained equal to those used in the individual treatments.

At the end of all treatments, the biomass samples were centrifuged to remove residual reagents and buffer components, frozen, and freeze-dried prior to analytical characterization. All treatments were performed in duplicate replicates. Statistical analyses were performed using R (Foundation for Statistical Computing, Vienna, Austria, version 4.3.1). Differences in PHA purity and HV content among purification treatments were evaluated by one-way analysis of variance (ANOVA), followed by Tukey’s honestly significant difference (HSD) post-hoc test for multiple pairwise comparisons. Statistical significance was accepted at *p* < 0.05.

### 2.6 PHA quantification and polymer characterisation

PHA content and monomeric composition (3HB and 3HV) of the obtained PHBV-rich biomass were analysed according to the procedure reported by (Braunegg et al., 1978). Briefly, 2–5 mg of dried biomass underwent acid-catalysed methanolysis to convert the polymeric 3-hydroxyacyl fractions into the corresponding methyl esters, which were subsequently quantified by gas chromatography. A volume of 1 μL of the organic phase was injected into a Thermofisher TRACE 1600 GC system fitted with a TG-5MS column. Nitrogen served as the carrier gas at a flow rate of 1.5 mL/min. The oven temperature was initially maintained at 100 °C for 1 min, then increased to 130 °C at a rate of 12 °C/min, followed by a further increase to 250 °C at 25 °C/min. The flame ionisation detector (FID) temperature was set at 320 °C. Quantification of 3HB and 3HV was carried out using a PHBV standard containing 12% (w/w) 3HV (Sigma-Aldrich), while benzoic acid was employed as the internal standard. The intracellular PHA content (%, w/w) was expressed as a percentage of mixed liquor suspended solids (MLSS).

The molecular weight parameters, including the weight-average molecular weight (Mw), number-average molecular weight (Mn), and polydispersity index (PDI = Mw/Mn), were determined after chemical and enzymatic treatments and also for the untreated biomass by extracting the polymer from dried biomass using chloroform and methanol, according to (Braunegg et al., 1978). Extraction was performed at 70 °C for 4 h, after which the solution was filtered and PHAs were precipitated by the addition of methanol at a ratio of 5 mL per mL of chloroform. The precipitated polymer was recovered by filtration, transferred onto glass Petri dishes, and dried overnight at room temperature to allow solvent evaporation. The purified polymer was subsequently characterised by gel permeation chromatography (GPC) using a system equipped with a JASCO PU-4180 pump, a guard column, two TSKgel® columns (G6000-HHR and GMHHR-H), a JASCO CO-4060 column oven, and a JASCO RI-4030 refractive index detector. Chloroform was used as the mobile phase at a flow rate of 1 mL/min. The column oven and detector temperatures were maintained at 40 °C and 35 °C, respectively. Calibration curves were established using polystyrene standards with molecular weights ranging from 0.013 to 3.05 × 10^6^ g/mol. Because polystyrene standards were used for calibration, the reported Mw and Mn values represent polystyrene-equivalent molecular weights and should therefore be considered relative rather than absolute molecular-weight values. Independent replicate polymer samples were not available for molecular weight determination; therefore, Mw, Mn and PDI are presented as descriptive values and were not subjected to inferential statistical analysis.

### 2.7 Chemical analyses

MLSS, MLVSS, and COD were determined in accordance with Standard Methods (APHA, 1998). For analyses of the soluble fraction, samples were first filtered through 0.45 μm membrane filters. Ammonia concentrations were measured by distillation followed by colourimetric determination using Nessler’s reagent (Irsa-Cnr et al., 2008), whereas soluble reactive phosphorus was analysed using a commercial test kit (Phosphate LCK348, Hach Lange GmbH). CAs were quantified by ion chromatography using a Dionex™ ICS-1100 system (Thermo Fisher Scientific) equipped with an IonPac ICE-AS1 column, according to the methodology described by Raposo et al. (2015). The concentrations of individual CAs, including acetic, propionic, butyric, isobutyric, pentanoic, isopentanoic, and hexanoic acids, were determined separately, while the total CA concentration was calculated as the sum of all quantified compounds.

PHA content and purity were calculated as the ratio between the total mass of 3HB and 3HV and the mass of dried biomass introduced into the analysis ( $m_{dw}$ ):

$$PHBV\left(\%\frac{w}{w}\right)=\frac{\left(m3HB+m3HV\right)}{m_{dw}}$$

The fraction of 3HV in the polymeric matrix was determined as:

$$3HV\left(\%\frac{w}{w}\right)=\frac{m3HV}{\left(m3HB+m3HV\right)}$$

PHA content refers to the PHA fraction of the untreated biomass, whereas PHA purity refers to the PHA fraction of the dried material recovered after downstream treatment. PHA recovery yield was not determined in this study.

The PHA storage yield (Y_PHA/CA_) was obtained as:

$$Y_{PHA/CA}\;\left(gCOD/gCOD\right)=\frac{\left({\Delta COD}_{PHA}\right)}{\left({-\Delta COD}_{CA}\right)}$$

Where stoichiometric conversion factors were: 1.67 g COD/g for 3HB and 1.92 g COD/g for 3HV.

Standard errors associated with all reported parameters were estimated using conventional error propagation equations.

### 2.8 Microbial community analysis

Changes in the bacterial community during the selection phase were evaluated to assess the enrichment of PHA-producing microorganisms. A total of four samples were analysed, corresponding to two samples from each of the two selected experimental batches (Batch 12 and Batch 14). For each batch, one sample consisted of the inoculum collected from the full-scale wastewater treatment plant in Verona, Italy, while the second sample was collected at the end of the selection phase in SBR1.

Two millilitres of each sample were centrifuged at maximum speed for 2 min, and the resulting pellet was sent to BMR Genomics (Padua, Italy) for 16S rRNA gene amplicon sequencing. DNA extraction was performed using the DNeasy® 96 PowerSoil® Pro QIAcube® HT Kit (Qiagen), followed by purification on the QIAcube HT system. The V4 hypervariable region of the 16S rRNA gene was amplified using universal primers targeting both bacteria and archaea (Takahashi et al., 2014). The resulting amplicons were purified using Thermolabile Exonuclease I (NEB). Sequencing libraries were prepared using the Nextera XT DNA Library Preparation Kit (Illumina). Subsequently, samples were normalised, pooled, and sequenced on an Illumina MiSeq platform.

Sequence quality assessment was performed using FASTQC, while taxonomic classification was conducted through the QIIME2 pipeline against the SILVA 132 and RDP reference databases. Taxonomic assignments obtained from the different databases were compared for quality control, whereas SILVA 132 was used for the analyses presented in this study. Microbial community analyses were performed at the genus level. Unknown and uncultured taxa were excluded, and the ten most abundant genera were considered for each sample.

## 3 Results and discussion

### 3.1 Feedstock preparation and carboxylic acid production

The substrate used for PHA production during Batch 12 was a synthetic CA mixture prepared directly in the carbon storage unit, as described in Section 2.2. For Batch 14, the feedstock was prepared in an external storage tank prior to feeding the acidogenic fermenter. The substrate consisted of a mixture of cattle slurry and maize silage, whose physicochemical characteristics are reported in Table 2. In both batches the substrate was prepared to obtain an initial concentration of around 10 g/L for COD and 0.5 and 0.1 g/L for nitrogen and phosphorus, respectively.

**TABLE 2:** Characterisation of the substrate produced by the external storage tank.

Parameter	Unit	Cattle slurry	Maize silage
TS	%	6.96	33.53
TVS	%	5.51	31.94
COD	g/Kg dry	883.43	772.14
NH_3_	gN/L	0.59	0.25
TKN	gN/kg dry	84.06	1.40

The use of an external preparation unit enabled homogenisation of the feedstock and ensured a stable supply to the fermentation reactor, thereby improving process stability, an important aspect for the operation of integrated agricultural biorefinery platforms (Atasoy et al., 2018). The acidogenic fermentation phase lasted 22 days and was operated under the conditions reported in Table 3.

**TABLE 3:** Operating parameters for the fermentation unit during Batch 14.

Parameters	Batch 14
Volume (L)	1700
HRT (d)	2.86
OLR (kgCOD·m^3^·d)	16
TS (%)	6
Feed volume (L/d)	594
Feed (VS) (KgVS/d)	35.7
Cow slurry (%)	50
Corn residues (%)	50
Cow slurry VS (KgVS)	14.1
Corn residues VS (KgVS)	14.1
Water (Kg/d)	294

Throughout the experimental period, the fermentation fluid exhibited an average total solids concentration of approximately 7%, of which about 55% corresponded to volatile solids, indicating a substantial biodegradable organic fraction available for acidification. Such solids concentrations are comparable to those commonly adopted for the acidogenic fermentation of livestock manure and agricultural residues and are considered suitable for promoting CA accumulation while avoiding excessive dilution of the fermentation broth (Strazzera et al., 2018).

The evolution of carboxylic acid production during the fermentation phase is shown in Figure 2. Total CA concentration increased steadily throughout the fermentation period, from 9.87 ± 0.01 gCOD/L on day 1–16.73 ± 0.72 gCOD/L on day 22, corresponding to a 69% increase in CA production. Simultaneously, pH remained relatively stable at 4.9–5.8, indicating the establishment of a stable acidogenic environment without the need for external pH control. Such mildly acidic conditions are known to suppress methanogenic activity while favouring CA accumulation (Atasoy et al., 2018). The final CA concentrations obtained in this study fall within the upper range reported for acidogenic fermentation of manure- and agro-residue-based substrates under mesophilic conditions, where values between 8 and 18 gCOD/L are commonly achieved depending on substrate composition, hydraulic retention time and pH management (Atasoy et al., 2018). The CA profile evolved considerably during the fermentation period. During the initial fermentation stage, only short-chain acids, namely, acetic, propionic and butyric acids, were detected. From day five onwards, valeric and caproic acids became measurable, indicating the establishment of more complex metabolic interactions, including chain elongation pathways (Wang et al., 2026). In particular, valeric acid increased continuously from 1.32 ± 0.02 to 3.56 ± 1.11 gCOD/L, while caproic acid progressively accumulated up to approximately 1.6–1.7 gCOD/L towards the end of the fermentation period. The present results closely resemble those previously reported by Pesante et al. (2026) at laboratory scale, demonstrating the successful transfer of the fermentation strategy from laboratory to pilot scale. In both studies, total CA concentrations stabilised within a comparable range (approximately 14–18 gCOD/L), indicating that the overall acidification efficiency was preserved despite the substantial increase in reactor volume and the integration of the process within an agricultural cooperative hosting a full-scale anaerobic digestion facility. Moreover, acetic and propionic acids remained the predominant fermentation products in both systems, suggesting that the main acidogenic pathways were maintained during scale-up. However, differences emerged for the longer-chain CAs. Compared with the laboratory-scale reactor, the pilot-scale process exhibited a more gradual and regular accumulation of valeric acid, whereas caproic acid concentrations remained lower and less variable. In contrast, the laboratory-scale system displayed larger temporal fluctuations and a more pronounced production of caproate. Such differences are expected during process transfer and can be attributed to changes in hydrodynamics, mixing efficiency, microbial stratification, substrate gradients and hydrogen partial pressure, all of which are known to influence chain elongation pathways (Zhou et al., 2018). Furthermore, the laboratory-scale experiments were conducted over considerably longer operational periods (up to approximately 100–150 days), whereas comparable CA concentrations were achieved here after only 22 days of operation. Although the two systems are not directly comparable due to differences in operating conditions, this observation suggests that process intensification can be achieved at pilot scale without compromising acidification performance. From an application perspective, the production of odd-carbon CAs is particularly advantageous for subsequent PHBV synthesis. Valerate constitutes an efficient precursor for the incorporation of 3-hydroxyvalerate (3HV) monomers into the polymer chain, thereby enabling the production of PHBV with improved flexibility and reduced brittleness without the addition of external chemical precursors (Ghysels et al., 2018; Hao et al., 2017). Therefore, the progressive accumulation of valeric acid observed during the second half of the fermentation period represents an additional advantage of the proposed process, as it may enhance the production of PHBV with improved mechanical properties without requiring external precursor addition. Overall, the acidogenic fermentation process generated a stable CA-rich stream with a composition suitable for subsequent MMC selection and PHBV accumulation, demonstrating the successful scale-up of the upstream stage of the integrated process.

**FIGURE 2:**
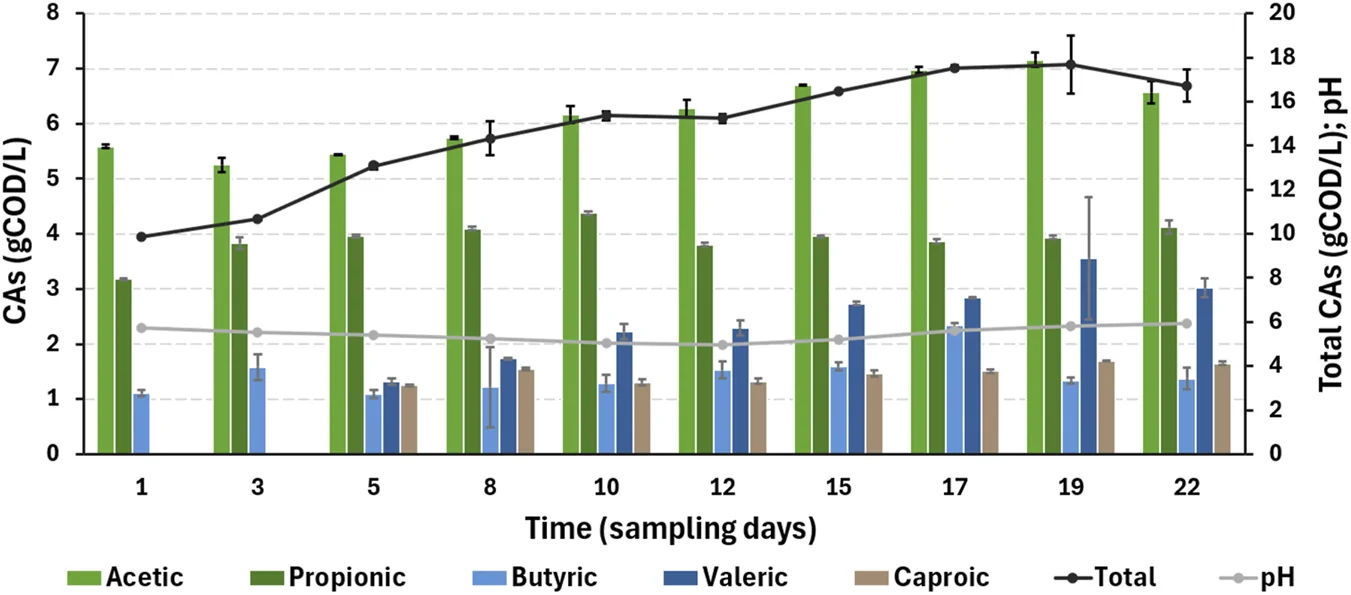
CAs produced by the fermentation unit during Batch 14.

### 3.2 Selection phase

The operational conditions adopted during the selection phase (Table 4) were designed to promote the enrichment of PHA-storing microorganisms by imposing a feast–famine regime, which is widely recognised as one of the most effective strategies for selecting MMCs capable of efficiently storing carbon as intracellular PHA (Johnson et al., 2009). Overall, both Batch 12 and Batch 14 were operated close to the target conditions defined in the Materials and Methods, indicating that a stable selective pressure was successfully maintained throughout the enrichment phase.

**TABLE 4:** Operational parameters for the selection SBR during Batch 12 and 14.

Parameters	Batch 12	Batch 14
Feast/famine ratio (F/F)	0.2	0.1
Cycle length (h)	6	12
Feast phase length (h)	1	1
Famine phase length (h)	5	11
N° cycles/d	4	2
Hydraulic retention time (d)	3	6
Reactor volume (L)	1040	800
Feed volume (L/cycle)	80	67
Feed volume (L/d)	320	133
Feedstock concentration (g COD/L)	9.7	9.7
COD_IN_ (Kg/cycle)	0.78	0.78
COD_IN_ (Kg/day)	3.1	1.3
Organic loading rate (Kg COD/m^3^d)	3.1	1.7
Temperature (°C)	25.0	25.0
pH	8.0–9.0	8.0–9.0

In both batches, the F/F ratio remained at or below the commonly recommended threshold of 0.20–0.25 (0.2 in Batch 12 and 0.1 in Batch 14). Maintaining a short feast phase relative to the total cycle duration is a key requirement for favouring microorganisms able to rapidly assimilate the available carbon source and subsequently rely on intracellular PHA reserves during the famine phase, thereby outcompeting non-storage organisms (Serafim et al., 2008). Although Batch 14 was operated with a lower F/F ratio, both operating strategies successfully imposed selective pressure towards storage specialists.

The cycle configuration differed substantially between the two campaigns. Batch 12 was operated with 6 h cycles (1 h feast and 5 h famine) and four cycles per day, whereas Batch 14 employed 12 h cycles (1 h feast and 11 h famine) and two cycles per day. Consequently, Batch 14 experienced a substantially longer famine period, potentially favouring the maintenance of PHA-storing populations. However, prolonged famine conditions may also increase endogenous respiration and reduce biomass activity if excessively extended, particularly when treating more heterogeneous feedstocks (Johnson et al., 2009).

Hydraulic retention times were set at 3 and 6 days for Batch 12 and Batch 14, respectively, while the organic loading rate (OLR) decreased from 3.1 to 1.7 kg COD m^-3^ d^-1^. Both values remained within the ranges commonly reported for pilot-scale MMC systems performing PHA selection (Valentino et al., 2017). The lower OLR adopted during Batch 14 was primarily a consequence of the longer cycle duration and reduced daily feeding volume and may have contributed to lower substrate availability throughout the selection process.

Temperature (25 °C) and pH (8.0–9.0) were maintained within the optimal ranges typically employed for MMC-based PHA production. In particular, slightly alkaline conditions have been associated with improved CA uptake and enhanced competitive advantage of PHA-storing microorganisms over non-storage populations (Serafim et al., 2008). The stability of these operational parameters throughout both campaigns indicates that any differences observed in subsequent accumulation and purification performances are unlikely to be attributable to deviations in reactor operation.

To complement process performance indicators and gain deeper insight into the effectiveness of the selection strategy, a 16S rRNA gene amplicon-based taxonomic analysis of the microbial communities was performed (Figure 3). Biomass samples were collected at the beginning and at the end of the selection phase for both experimental batches, allowing the evaluation of the effect of feast–famine enrichment on the mixed microbial cultures. Integrating microbial community characterisation within the selection phase enables the direct linkage of operational conditions with microbial adaptation and the enrichment of microorganisms potentially involved in PHA storage. While operating conditions provide indirect evidence of successful selection, microbial community analysis offers a mechanistic understanding of how these conditions shape community composition and ultimately influence process performance.

**FIGURE 3:**
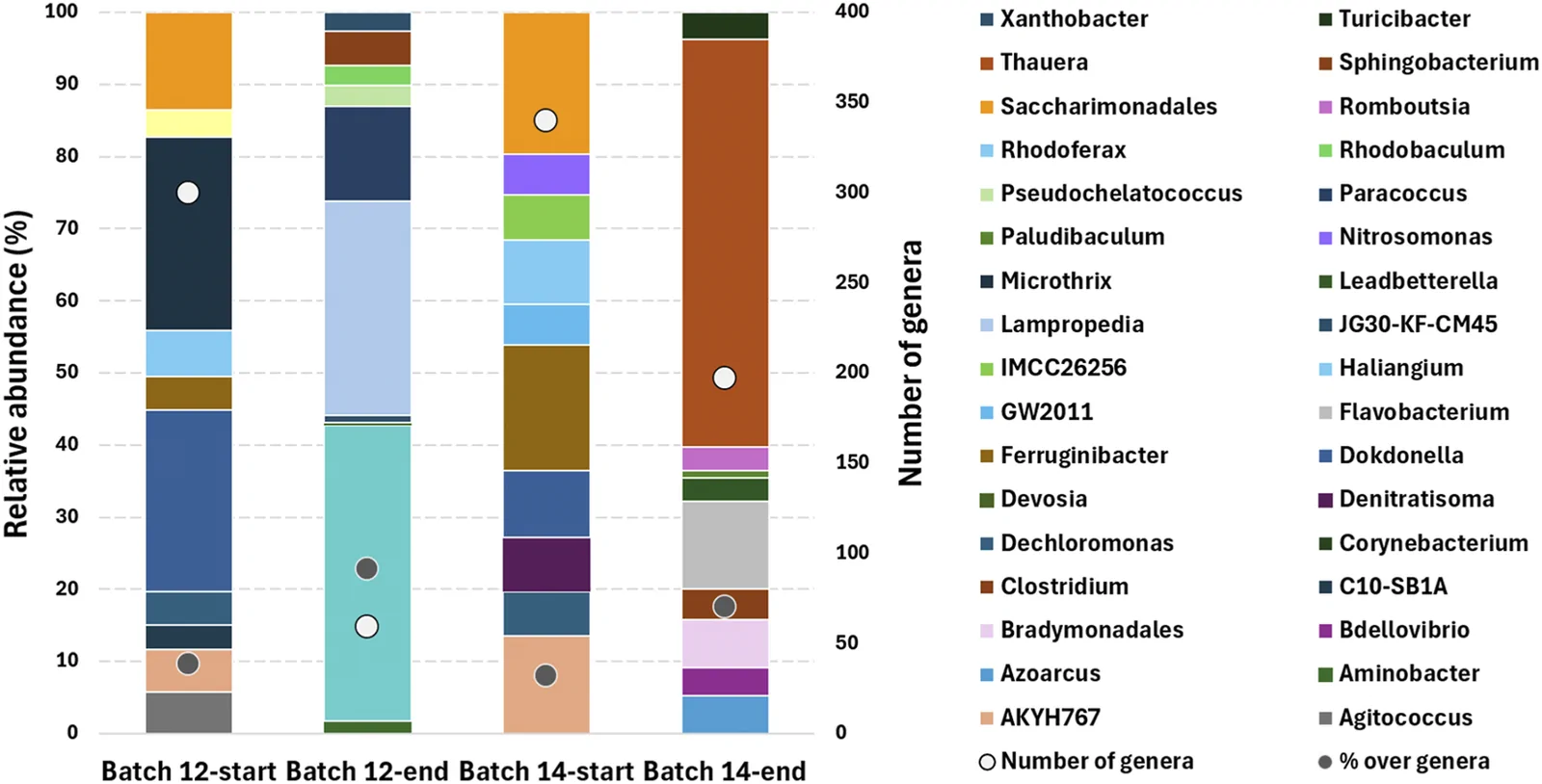
Bacterial community analysis at the genus level in the selection bioreactor for Batch 12 and 14. The ten most abundant genera per sample were considered, with their cumulative abundance shown by the dark grey marker line. The light grey line represents the total number of genera per sample.

The inocula derived from activated sludge exhibited highly diverse bacterial communities, comprising 300 and 340 detected genera in Batch 12 and Batch 14, respectively. Following selection, richness decreased substantially to 59 genera in Batch 12 and 197 genera in Batch 14, while the relative abundance represented by the ten most abundant genera increased from 38.6% to 91.3% and from 32.0% to 70.4%, respectively. Such a reduction in microbial diversity is a common feature of MMC selection processes, where the repeated imposition of feast–famine cycles progressively favours microorganisms capable of rapidly storing carbon as intracellular polymers (Serafim et al., 2008; Johnson et al., 2009). These results confirm that aerobic dynamic feeding successfully promoted the enrichment of specialised microbial populations adapted to rapid CA uptake and intracellular carbon storage. However, the extent of enrichment differed markedly between the two batches. Batch 12, fed with a synthetic CA mixture, exhibited a stronger reduction in diversity and a higher dominance of a limited number of taxa than Batch 14. This suggests that the synthetic substrate imposed a more stringent selective pressure, whereas the fermented agricultural feedstock likely provided a broader range of organic compounds and micronutrients capable of sustaining a more diverse microbial community, despite successful enrichment towards PHA-storing populations (Albuquerque et al., 2010). Furthermore, unlike the synthetic feedstock used in Batch 12, the real fermented feedstock employed in Batch 14 was neither sterilised nor filtered prior to feeding. Consequently, residual microorganisms introduced with the feedstock may also have contributed to community assembly during the selection phase, increasing the observed taxonomic diversity alongside the effect of the imposed feast–famine regime (Valentino et al., 2017). Consistently, Batch 14 retained a higher residual diversity at the end of the selection phase, which may also partially explain the lower purification efficiencies observed later during downstream processing, suggesting that biomass composition can influence not only PHA accumulation but also subsequent extraction and purification performances.

A complete restructuring of community composition was observed in both batches, as none of the dominant genera present in the inocula remained among the dominant taxa after enrichment, indicating that feast–famine operation selected specific functional groups rather than promoting the growth of initially abundant microorganisms. In Batch 12, the selected community was dominated by *Corynebacterium* (41.0%), *Lampropedia* (29.7%) and *Paracoccus* (13.1%), which together accounted for approximately 84% of the total abundance. In Batch 14, *Thauera* became the predominant genus, representing 56.5% of the community, followed by *Flavobacterium* (12.2%). Several of these genera, particularly *Thauera* and *Paracoccus*, have previously been associated with efficient PHA accumulation under feast–famine conditions and are frequently reported among dominant taxa in MMC systems treating CA-rich substrates (Lemos et al., 2008; Johnson et al., 2009). *Corynebacterium* has also been reported as a potential PHA-producing genus, although its role in MMC systems remains less extensively documented than that of classical PHA accumulators (Koller et al., 2009). Moreover, *Lampropedia* has recently emerged as a recurrent dominant genus in pilot-scale MMC systems operated under aerobic dynamic feeding, including previous studies performed on the same platform (Magonara et al., 2025; Pesante et al., 2026), while *Thauera, Paracoccus* and, to a lesser extent, *Flavobacterium* have all been detected in mixed-culture PHA-producing consortia treating waste-derived carbon sources (Valentino et al., 2017; Albuquerque et al., 2010). Collectively, these observations indicate that feast–famine selection consistently enriches functionally relevant PHA-storing populations, although the dominant taxa may vary according to feedstock characteristics and operating conditions.

The microbial community observed in Batch 14 also highlights the potential synergy of MMC with the agricultural feedstock for efficient PHA production. Under the feast–famine regime, *Thauera* became the dominant genus, accounting for 56.5% of the community, while *Flavobacterium* represented a further 12.2%. The predominance of *Thauera*, typically recognised as a denitrifying bacterium in wastewater treatment systems (Lemos et al., 2008), is however consistent with previous studies showing its recurrent enrichment in PHA-producing MMCs operated under feast–famine conditions and its demonstrated capacity for PHA storage from Cas (Sruamsiri et al., 2020). The presence of *Flavobacterium*, known for its versatile enzymatic machinery (Yang et al., 2025) may be relevant to the utilisation of the more complex organic fraction of agricultural-derived substrates, as members of this genus have been reported to be able to degrade high-molecular-weight and recalcitrant organic compounds in lignocellulose-degrading microbial communities (Cortes-Tolalpa et al., 2017). Thus, the observed community shift suggests that the heterogeneous agricultural feedstock can support a consortium in which different microbial populations may contribute to substrate conversion and PHA accumulation. Further optimisation of feedstock composition, feast–famine conditions and nutrient availability could therefore help steer community structure and PHA productivity.

Interestingly, the enrichment patterns observed at pilot scale differed from those previously reported in laboratory-scale studies performed on the same platform, where *Neomegalonema* and *Lampropedia* were identified as the dominant genera after selection (Magonara et al., 2025; Pesante et al., 2026). Nevertheless, the recurrent detection of recognised PHA-associated genera such as *Paracoccus* and *Thauera* suggests that similar functional traits were selected despite differences in taxonomic composition. This observation supports the concept of functional redundancy in MMC systems, whereby distinct microbial communities can achieve comparable PHA production performances provided that storage-associated metabolic capabilities are maintained (Reis et al., 2011).

Overall, these results demonstrate that the imposed operating conditions successfully selected specialised PHA-storing MMCs in both campaigns, while simultaneously highlighting how feedstock characteristics can shape microbial community structure and influence downstream process performance. By directly linking process performance with microbial community dynamics, this integrated process-to-microbiome approach strengthens the interpretation of the pilot-scale results and highlights the importance of microbial ecology for the development of scalable MMC-based PHBV production processes.

### 3.3 Accumulation phase

The accumulation phase was performed in SBR2 under fed-batch conditions using a multi-spike feeding strategy, as described in the Materials and Methods section. Selected biomass from SBR1, at a typical VS concentration of around 2.0–3.0 g/L, was transferred to SBR2 and subjected to repeated substrate additions to maintain a target concentration of 0.5 g CA_COD_ L^−1^. Dissolved oxygen (DO) monitoring was used to identify substrate depletion and regulate the feeding sequence: DO concentration remained below 1 mg/L during the initial phase of the cycle, coinciding with CA addition and high metabolic activity, and progressively increased to approximately 6 mg/L as the CAs were depleted and intracellular PHA accumulation proceeded.

Compared with the target operating conditions described in the Materials and Methods, minor adjustments were implemented during the pilot campaigns to account for differences in reactor working volumes between the two experimental setups. In particular, the initial volume was reduced from 1080 L in Batch 12–800 L in Batch 14, and spike volumes were proportionally adapted from 12.7 to 9.4 L while maintaining six feeding events at hourly intervals. These adjustments maintained the target CA concentration while accommodating differences in biomass availability and reactor operation. Maintaining a constant substrate concentration through multiple feeding pulses is a well-established strategy to promote sustained PHA synthesis while preventing substrate overloading and excessive microbial growth (Johnson et al., 2009; Serafim et al., 2008; Reis et al., 2011).

When operating with synthetic substrate, as in Batch 12, the biomass concentration at the end of the accumulation phase reached approximately 2.5–3.0 g VS/L, with a PHA content of 26% and an HV content of 26% (Table 5). Under these conditions, the volumetric PHA productivity was 0.14 g PHA/L/h, while the Y_PHA/CA_ was approximately 0.87 gPHA/gCOD consumed. These values are comparable to those reported by Valentino et al. (2019) for a pilot-scale reactor operated with synthetic acetate as the carbon source. In Batch 14, where real fermentate was used as the carbon source, the biomass concentration at the end of the accumulation phase ranged from 3.0 to 3.5 g VS/L, partly depending on the presence of suspended solids in the fermentate. The PHA content was approximately 27%–30%, with an HV content of 14% (Table 5). The resulting volumetric PHA productivity (0.15 gPHA/L/h) was comparable to that obtained with the synthetic substrate. However, based on the amount of COD available in each batch, the Y_PHA/CA_ was approximately 0.53 g PHA/g COD consumed, markedly lower than that observed for Batch 12. These values are comparable to those reported by Gottardo et al. (2022) for PHA production using CA derived from food-processing waste.

**TABLE 5:** Summary of all pilot-scale PHA accumulation campaigns performed during process optimisation, including feedstock type, polymer composition, downstream purification strategy and PHA production. NaOH = sodium hydroxide, HAc = acetic acid.

Batch	Start date	End date	Feedstock	PHA end accumulation (%)	HV end accumulation (%)	Purification type	PHA end purification (%)	HV end purification (%)	Recovered PHBV-rich biomass (g)
1	27/02/2024	19/03/2024	Synthetic	8.4	50.1	NaOH	2.7	17.5	800
2	02/04/2024	23/04/2024	Synthetic	41.3	24.6	NaOH	6.5	20.4	N/A
3	24/04/2024	13/06/2024	Synthetic	36.3	27.4	NaOH	17.0	50.0	143
4	13/06/2024	25/07/2024	Synthetic	3.0	51.0	NaOH	5.0	40.0	973
5	26/07/2024	28/08/2024	Synthetic	18.0	16.0	NaOH	2.0	N/A	524
6	29/08/2024	09/10/2024	Synthetic	5.0	8.0	NaOH	2.0	N/A	906
7	13/11/2024	28/11/2024	Synthetic	30.0	16.0	NaOH	11.0	16.0	850
8	28/11/2024	12/12/2024	Synthetic	35.0	18.0	NaOH	10.0	22.0	686
9	12/02/2025	25/02/2025	Synthetic	33.0	16.0	NaOH	22.0	17.0	650
10	27/02/2025	10/03/2025	Synthetic	33.0	17.0	NaOH + HAc	61.0	16.0	620
11	28/03/2025	07/04/2025	Synthetic	29.0	16.0	NaOH + HAc	56.0	16.0	755
12	14/05/2025	27/05/2025	Synthetic	26.0	26.0	NaOH + HAc	58.0	23.0	1158
13	19/06/2025	01/07/2025	Synthetic	16.0	27.0	NaOH + HAc	32.0	22.0	108
14	22/10/2025	11/11/2025	Real	30.0	14.0	NaOH + HAc	37.0	14.0	1033
15	12/12/2025	18/12/2025	Real	27.0	16.0	NaOH + HAc	23.0	17.0	989

HV, values obtained at very low PHA, contents should be considered indicative, as the low amount of polymer available for analysis may increase the uncertainty associated with the determination.

Following the accumulation phase, the biomass was concentrated by flocculation, chemically quenched, and transferred for downstream purification.

Table 5 summarises all pilot-scale accumulation campaigns performed between February 2024 and December 2025. These experiments represent a progressive optimisation process carried out over multiple operational runs; however, the present study focuses primarily on the two campaigns (Batch 12 and Batch 14) which were selected for detailed analysis as they represent the optimised synthetic- and real-feedstock scenarios discussed throughout the manuscript. The previous campaigns are nevertheless reported to illustrate process variability and the progressive improvement achieved during pilot-scale operation.

Substantial variability was observed among the pilot campaigns, particularly during the early stages. PHA contents at the end of the accumulation phase ranged from 3.0% to 41.3%, while HV contents varied between 8.0% and 51.0%. Similarly, purification efficiencies after NaOH treatment remained generally low, ranging from 2% to 22%, indicating that downstream processing represented one of the main bottlenecks of the overall process. This variability is commonly observed in MMC systems, where process performance is strongly influenced by operational conditions, biomass composition and substrate characteristics (Reis et al., 2011; Serafim et al., 2008). A clear improvement was observed after the introduction of the sodium hydroxide-acetic acid purification protocol. Campaigns 10 to 12 consistently achieved higher purification efficiencies (56%–61%) compared with all previous NaOH-only treatments, confirming the benefits already highlighted during laboratory-scale optimisation. This improvement was particularly evident in Batch 12, which reached a PHA purity of 58.0% ± 0.1% after purification while maintaining an HV content of 23.0% ± 0.7%. These results are fully consistent with the laboratory-scale optimisation experiments, supporting the successful transfer of the selected protocol from laboratory to pilot scale under synthetic-feedstock conditions.

The final real-feedstock campaign (Batch 14) achieved a PHA content of 30.0% ± 0.2% at the end of the accumulation phase, comparable to several synthetic-feedstock campaigns, while maintaining an HV content of 14.0% ± 1.0%. However, downstream purification reached a concentration of PHA of only 37.0% ± 0.1%, substantially lower than the values obtained for Batch 12. Unlike Batch 12, no laboratory-scale optimisation was specifically performed for this feedstock, and the sodium hydroxide-acetic acid protocol was directly transferred from synthetic to real conditions. The lower purification efficiency observed in Batch 14 therefore highlights the limitations associated with translating laboratory-optimised protocols to more complex substrates without prior feedstock-specific optimisation. The lower performance of Batch 14 may also be explained by the intrinsic characteristics of real feedstocks, which are typically more heterogeneous than synthetic substrates and may generate biomass containing higher proportions of suspended solids, recalcitrant organic matter and a more complex extracellular matrix. These characteristics can influence not only microbial selection and PHA accumulation but also subsequent downstream processing. Indeed, the microbial community analysis presented in Section 3.2 showed that Batch 14 retained a higher residual diversity after selection than Batch 12, suggesting that feedstock composition (and microbial contamination) influences the overall process chain from microbial ecology to polymer recovery. Similar observations have been reported in previous studies showing that complex waste-derived substrates support more diverse microbial communities and increase process variability compared with synthetic substrates (Albuquerque et al., 2010; Valentino et al., 2017).

Overall, the results demonstrate a progressive improvement in pilot-scale performance throughout the optimisation campaigns and confirm that laboratory-scale optimisation performed on synthetic-feedstock biomass can be successfully translated to pilot scale under comparable operating conditions. Conversely, real feedstocks require dedicated optimisation strategies due to their greater microbial and compositional complexity. Nevertheless, both final campaigns successfully produced PHBV with HV contents within ranges commonly reported for flexible and processable copolymers, further supporting the feasibility of producing value-added biopolymers from heterogeneous waste streams under pilot-scale conditions (Valentino et al., 2017; Serafim et al., 2008). The effect of feedstock characteristics on downstream recovery is further discussed in the following section, where the extraction and purification performances obtained for the two representative campaigns are analysed in detail.

### 3.4 PHA extraction and purification

Laboratory-scale extraction and purification of MMC-derived biomass showed distinct performances across chemical, enzymatic and combined chemical–enzymatic treatments (Figure 4; Table 6). These optimisation experiments were performed using a representative biomass sample obtained from the pilot-scale Batch 12 (synthetic feedstock) and subsequently used to select the downstream purification strategy for the bulk pilot-scale biomass.

**FIGURE 4:**
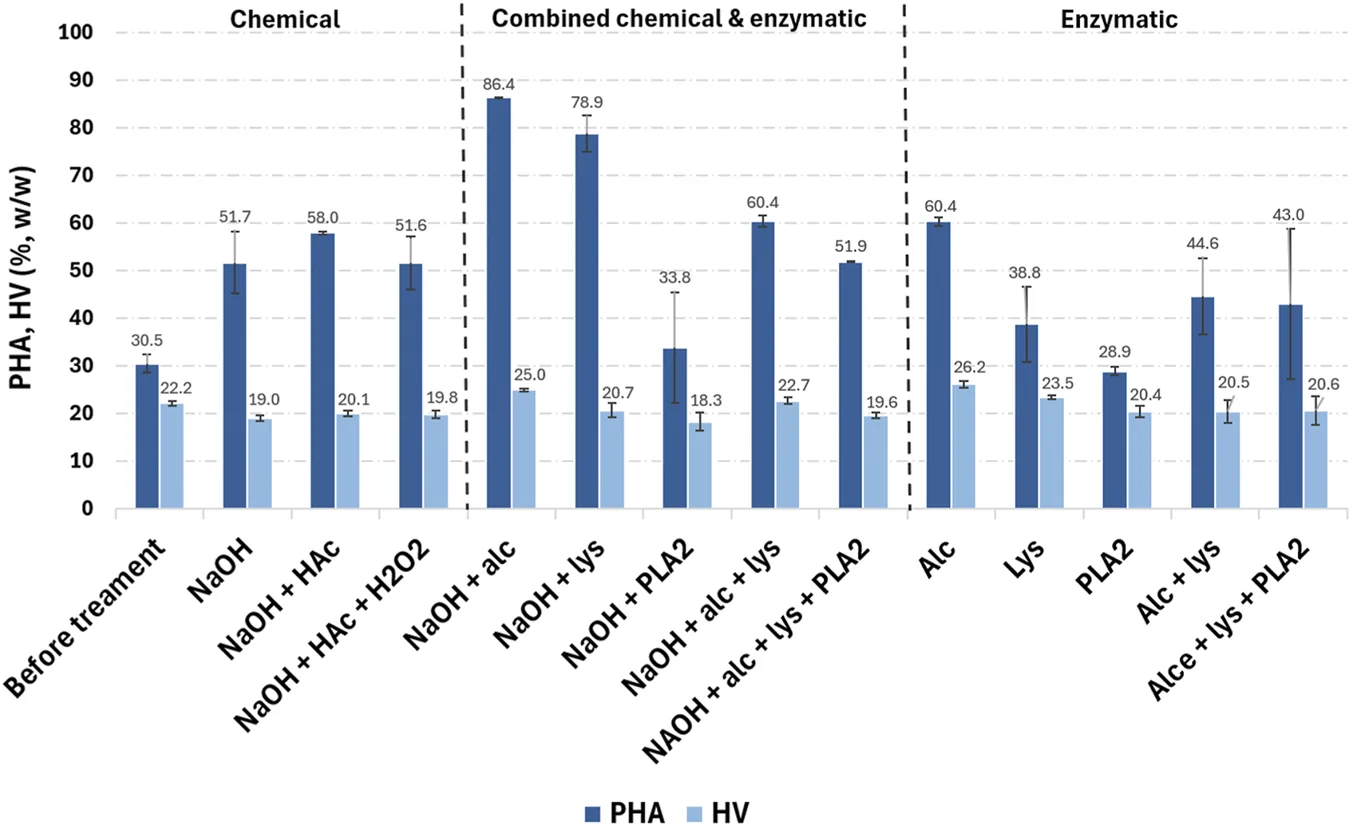
PHA purity and 3-hydroxyvalerate (HV) content of the biomass obtained from Batch 12 after chemical, enzymatic, and combined chemical–enzymatic purification treatments at laboratory scale. The sample labeled “before treatment” represents the raw biomass prior to any treatment. Different lowercase letters indicate statistically significant differences between treatments according to Tukey’s HSD test. NaOH = sodium hydroxide, HAc = acetic acid, H_2_O_2_ = hydrogen peroxide, alc = alcalase, lys = lysozyme, PLA2 = phospholipase A2.

**TABLE 6:** Molecular characteristics of the PHA recovered from Batch 12 after chemical, enzymatic, and combined chemical–enzymatic purification treatments at laboratory scale. The sample labeled “before treatment” represents the raw biomass before any treatment.

Treatment type	Mw (kDa)	Mn (kDa)	PDI
Before treatment	438.65	149.45	2.94
NaOH	521.35	244.66	2.13
NaOH + HAc	560.19	210.30	2.66
NaOH + HAc + H_2_O_2_	411.27	102.76	4.00
NaOH + alc	411.55	170.74	2.41
NaOH + lys	395.22	120.66	2.18
NaOH + PLA2	271.54	128.80	2.12
NaOH + alc + lys	337.77	100.33	3.37
NAOH + alc + lys + PLA2	365.49	165.30	2.21
Alc	491.61	167.56	2.93
Lys	392.85	131.53	2.99
PLA2	359.06	225.86	1.59
Alc + lys	423.93	179.68	2.36
Alc + lys + PLA2	268.94	131.85	2.04

Mw = weight-average molecular weight, Mn = number-average molecular, PDI = polydispersity index.

Among the chemical protocols, sodium hydroxide (NaOH) – alone or with acetic acid (HAc) or hydrogen peroxide (H_2_O_2_) – increased PHA purity from 30.5% in the untreated biomass to 51.7%, 58.0% or 51.6%, respectively, representing a statistically significant improvement compared with the untreated biomass (Tukey post-hoc test, *p* = 0.0268). However, the differences among the three chemical treatments were not statistically significant (*p* > 0.5), indicating that the addition of HAc or H_2_O_2_ did not further increase the purity. NaOH and NaOH and NaOH-HAc yielded Mw values of 521.4 kDa and 560.2 kDa, respectively, compared with 438.7 kDa for the crude polymer. The PDI was 2.13 after NaOH treatment, compared with 2.94 in the crude polymer. As previously reported for MMC-derived PHAs the differences in Mw observed among the untreated and treated samples may reflect the removal of low-molecular-weight oligomers and degraded polymer fractions that bias Mw determination in crude extracts, rather than to polymer chain extension, which is not expected under mild alkaline or acidic conditions (Lorini et al., 2021; Zou et al., 2023; Pagliano et al., 2021). In contrast, the NaOH–HAc–H_2_O_2_ sequence yielded an Mw of 411.3 kDa and a PDI of 4.00. These values were lower and higher, respectively, than those observed after NaOH–HAc treatment; however, given that molecular-weight measurements were performed as single determinations, these differences are reported descriptively and cannot be interpreted as statistically demonstrated changes **.**(Koller and Mukherjee, 2022; Mannina et al., 2020). The HV content remained relatively stable following the chemical treatments. Only the NaOH purification protocol produced a statistically significant reduction in HV compared with the untreated biomass from 22.2% to 19.1% (*p* = 0.0205), while no other significant difference was observed.

Enzymatic treatments produced more variable results. We compared chemical treatments to single-enzyme treatments using a 1-way ANOVA. Alcalase alone achieved a PHA purity of 60.4%, comparable to the NaOH–HAc protocol (p > 0.5), and represented a statistically significant improvement compared with the untreated biomass (*p* = 0.0069). This finding confirms that proteolytic digestion can efficiently remove non-PHA biomass (Pagliano et al., 2021). Conversely, lysozyme and phospholipase A2 showed limited effectiveness, as the PHA content after treatment (38.8% and 28.9%, respectively) did not differ from untreated biomass (p > 0.5), reflecting their narrow substrate specificity and the heterogeneous composition of MMC biomass. Combining enzymes did not improve purification: the alcalase–lysozyme treatment reached only 44.6% purity, while the addition of PLA2 further reduced performance (43.0%). This behaviour is likely explained by the different optimal pH and temperature requirements of the enzymes, which constrain catalytic efficiency when applied sequentially under shared operating conditions, a limitation commonly reported for multi-enzyme systems (Siedentop et al., 2021). Moreover, enzymatic hydrolysis alone may only partially disrupt heterogeneous biomass matrices without completely removing non-PHA components (Van Dyk and Pletschke, 2012). Although enzyme-based recoveries above 90% have been reported, these generally refer to laboratory-grown pure cultures and involve additional pretreatment steps, making direct comparison with MMC systems difficult (Mannina et al., 2020; Pagliano et al., 2021). To the best of our knowledge, this is one of the first studies to systematically evaluate enzymatic purification strategies for pilot-scale MMC-derived PHA, whereas previous enzymatic recovery efforts have focused primarily on pure cultures produced at laboratory scale or have used enzymes only as auxiliary steps (Kapritchkoff et al., 2006). Interestingly, the different enzymatic treatments were also associated with differences in polymer molecular characteristics. Alcalase alone yielded an Mw of 491.6 kDa, while Mw values of 392.9 and 359.1 kDa were obtained after lysozyme and PLA2 treatments, respectively**.**The PLA2 treatment, however, exhibited the lowest PDI (1.59) among all enzymatic treatments, suggesting a more homogeneous polymer fraction despite its limited purification efficiency.

The combined chemical–enzymatic treatments achieved the highest PHA purities overall, with some treatments (NaOH-alc and NaOH-lys) producing statistically significant improvements compared to the untreated biomass. NaOH followed by alcalase reached 86.4% purity, the highest among all tested protocols, while NaOH followed by lysozyme achieved 78.9%. Both treatments resulted in statistically significant increases in PHA purity compared with the untreated biomass (*p* < 0.01), with NaOH–alcalase also showing significantly higher purity than NaOH and alcalase alone (*p* < 0.01). No significant differences were highlighted between the two combined protocols NaOH-alc and NaOH-lys. However, the reported improvements were associated with Mw values lower than that observed for the NaOH–HAc treatment (e.g., 411.6 vs. 560.2 kDa for NaOH–alcalase and NaOH–HAc, respectively), and the NaOH–alcalase–lysozyme sequence yielded the lowest observed Mw (337.8 kDa) and a PDI of 3.37. Such trade-offs between polymer purity and molecular weight preservation are widely recognised in PHA downstream processing (Mannina et al., 2020; Koller and Mukherjee, 2022). Across all treatments, the HV fraction remained within the desired range (20%–30%), although some statistically significant differences were detected among individual treatments, indicating that, overall, the extraction protocols did not significantly alter PHBV copolymer composition. Although the NaOH–alcalase sequence achieved the highest puritythe additional enzyme requirements and associated costs represent an important consideration for scaling up enzymatic downstream processing. Previous techno-economic assessments have highlighted the significant contribution of material inputs and downstream operations to PHA recovery costs, with chemical cell-lysis approaches generally showing lower estimated costs than solvent-based alternatives (Pagliano et al., 2021). Enzymatic recovery therefore remains attractive from a purification-performance perspective, but its economic feasibility at pilot and industrial scale depends on enzyme dosage and cost (Koller and Mukherjee, 2022; Kapritchkoff et al., 2006). Accordingly, the NaOH–HAc protocol was selected as the best compromise between purification efficiency, polymer preservation, operational simplicity and expected scalability. The selected protocol was subsequently applied to the pilot-scale biomasses produced during Batch 12 and Batch 14 (Table 7). Batch 12 showed excellent agreement with the laboratory-scale optimisation, with PHA purity increasing from 26% to 58%, while the HV fraction was largely preserved (26%–23%). The recovered polymer also exhibited a Mw of 560.2 kDa and a moderate polydispersity index (PDI = 2.66), consistent with the laboratory-scale optimisation results and confirming that the selected protocol effectively preserved polymer quality during pilot-scale implementation. These results confirm the robustness and reproducibility of the selected protocol when transferred from laboratory to pilot scale under comparable operating conditions. Conversely, Batch 14 exhibited a considerably lower purification efficiency, with PHA purity increasing only from 30% to 37%, although the HV content remained unchanged (14%). Despite the lower purification efficiency, the recovered polymer showed an Mw of 370.7 kDa) together with a lower PDI (2.04) than Batch 12, indicating that polymer chain integrity was largely preserved despite the more complex biomass matrix. Unlike Batch 12, this biomass was generated using a real waste-derived feedstock, which is intrinsically more heterogeneous and contains larger amounts of suspended solids, inorganic particles and recalcitrant organic matter. Moreover, no laboratory-scale optimisation was performed specifically on this biomass before pilot-scale application. The lower purification efficiency most likely reflects both the greater complexity of the biomass and the direct transfer of a protocol optimised on synthetic-feedstock biomass. Similar observations have been reported for MMC-based PHA production, where biomass composition and extracellular polymeric substances strongly influence downstream recovery efficiency and often require feedstock-specific optimisation (Valentino et al., 2017; Morgan-Sagastume et al., 2015).

**TABLE 7:** Characteristics of the PHBV obtained from Batch 12 and 14 after accumulation and after extraction and purification with sodium hydroxide (NaOH) and acetic acid (HAc).

Parameters	Unit	Batch 12	Batch 14
PHA end of accumulation phase	% w/w, ±SD	26.0 ± 0.1	30.0 ± 0.2
HV end of accumulation phase	% w/w, ±SD	26.0 ± 0.8	14.0 ± 1.0
PHA after extraction/purification with NaOH + HAc	% w/w, ±SD	58.0 ± 0.1	37.0 ± 0.1
HV after extraction/purification with NaOH + HAc	% w/w, ±SD	23.0 ± 0.7	14.0 ± 0.6
Mw	kDa	560.19	370.734
Mn	kDa	210.19	181.632
PDI	kDa/kDa	2.66	2.04

Acc. = accumulation phase, SD = standard deviation, Mw = weight-average molecular weight, Mn = number-average molecular, PDI = polydispersity index.

Overall, these results demonstrate that laboratory-scale optimisation can be successfully translated to pilot scale when biomass characteristics remain comparable, as observed for Batch 12. In contrast, the lower performance obtained for Batch 14 highlights the need for dedicated optimisation when processing biomasses produced from real waste streams. Nevertheless, the preservation of HV content in both batches confirms that the selected purification protocol maintained PHBV copolymer composition despite differences in feedstock complexity.

## 4 Conclusion

This study demonstrated the feasibility of producing PHBV from agri-food residues at pilot scale using mixed microbial cultures within an integrated biorefinery platform encompassing the complete production chain, from acidogenic fermentation to biomass recovery and downstream purification. Although the pilot-scale implementation required progressive optimisation over multiple operational campaigns, the process proved sufficiently robust to produce PHBV from both synthetic and real agricultural feedstocks, highlighting both the potential and the practical challenges associated with scaling up MMC-based bioprocesses.

Laboratory-scale optimisation identified the NaOH–HAc protocol as the best compromise between polymer purity, molecular weight preservation, operational simplicity and process economics. Although the polymer purities remained lower than those typically achieved with pure cultures, the PHBV produced contained 14%–26% HV, a composition that may contribute to enhanced flexibility compared with PHB and supports the potential of the recovered material for agricultural applicationsThe thermal, mechanical and biodegradation properties of the recovered materials are currently being investigated within the AgriLoop project (https://www.agriloop-project.eu/) and will further support the assessment of their suitability for applications such as plant pots and vineyard clips. Overall, this work provides one of the few integrated pilot-scale demonstrations of MMC-based PHBV production from agricultural residues and contributes to advancing the industrial implementation of sustainable PHA biorefineries.

## Data Availability

The data presented in the study are accessible using doi https://doi.org/10.57745/PA4ZCV

## References

[B1] Albuquerque M. Torres C. Reis M. (2010). Polyhydroxyalkanoate (PHA) production by a mixed microbial culture using sugar molasses: effect of the influent substrate concentration on culture selection. Water Res. 44, 3419–3433. 10.1016/j.watres.2010.03.021 20427069

[B2] APHA (1998). Standard Methods for the Examinations of Water and Wastewater, Washington, DC: American Public Health Association

[B3] Atasoy M. Owusu-Agyeman I. Plaza E. Cetecioglu Z. (2018). Bio-based volatile fatty acid production and recovery from waste streams: current status and future challenges. Bioresour. Technology, 268, 773–786. 10.1016/j.biortech.2018.07.042 30030049

[B4] Bengtsson S. Hallquist J. Werker A. Welander T. (2008). Acidogenic fermentation of industrial wastewaters: effects of chemostat retention time and pH on volatile fatty acids production. Biochem. Eng. J. 40, 492–499. 10.1016/j.bej.2008.02.004

[B5] Braunegg G. Sonnleitner B. Lafferty R. (1978). A rapid gas chromatographic method for the determination of poly-β-hydroxybutyric acid in microbial biomass. Eur. J. Appl. Microbiol. Biotechnol. 6, 29–37. 10.1007/BF00500854

[B6] Chen G.-Q. Patel M. K. (2012). Plastics derived from biological sources: present and future: a technical and environmental review. Chem. Reviews 112, 2082–2099. 10.1021/cr200162d| 22188473

[B7] Choi J. Lee S. Y. (1999). Factors affecting the economics of polyhydroxyalkanoate production by bacterial fermentation. Appl. Microbiol. Biotechnol. 51, 13–21. 10.1007/s002530051357

[B8] Cortes-Tolalpa L. Salles J. F. VAN Elsas J. D. (2017). Bacterial synergism in lignocellulose biomass degradation–complementary roles of degraders as influenced by complexity of the carbon source. Front. Microbiology 8, 1628. 10.3389/fmicb.2017.01628 29067002 PMC5641323

[B9] Filippou K. Bouzani E. Kora E. Ntaikou I. Papadopoulou K. Lyberatos G. (2025). Polydroxyalkanoates production from simulated food waste condensate using mixed microbial cultures. Polymers 17, 2042. 10.3390/polym17152042 40808091 PMC12349330

[B10] Ghysels S. Mozumder M. S. I. De Wever H. Volcke E. I. Garcia-Gonzalez L. (2018). Targeted poly (3-hydroxybutyrate-co-3-hydroxyvalerate) bioplastic production from carbon dioxide. Bioresour. Technology 249, 858–868. 10.1016/j.biortech.2017.10.081 29136942

[B11] Gontard N. Shi A. Angellier-Coussy H. Aouf C. Bolzonella D. Broeze J. (2026). Circular agriculture design: integrated biorefinery to convert agri-residues into novel opportunities. Environ. Technol. and Innovation 43, 104984. 10.1016/j.eti.2026.104984

[B12] Gottardo M. Bolzonella D. Tuci G. A. Valentino F. Majone M. Pavan P. (2022). Producing volatile fatty acids and polyhydroxyalkanoates from foods bt-products and waste: a review. Bioresourse Technol. 361, 127716. 10.1016/j.biortech.2022.127716 35926558

[B13] Hao J. Wang X. Wang H. (2017). Overall process of using a valerate-dominant sludge hydrolysate to produce high-quality polyhydroxyalkanoates (PHA) in a mixed culture. Sci. Rep. 7, 6939. 10.1038/s41598-017-07154-3 28761106 PMC5537348

[B14] Irsa-Cnr A. Rome I. Istituto di Ricerca S. A. Jacquel N. Lo C.-W. Wei Y.-H. (2008). Isolation and purification of bacterial poly (3-hydroxyalkanoates). Biochem. Engineering Journal, 39, 15–27. 10.1016/j.bej.2007.11.029

[B15] Johnson K. Jiang Y. Kleerebezem R. Muyzer G. VAN Loosdrecht M. C. (2009). Enrichment of a mixed bacterial culture with a high polyhydroxyalkanoate storage capacity. Biomacromolecules 10, 670–676. 10.1021/bm8013796 19193058

[B16] Kapritchkoff F. M. Viotti A. P. Alli R. C. Zuccolo M. Pradella J. G. Maiorano A. E. (2006). Enzymatic recovery and purification of polyhydroxybutyrate produced by Ralstonia eutropha. J. Biotechnology 122, 453–462. 10.1016/j.jbiotec.2005.09.009 16253372

[B17] Koller M. Mukherjee A. (2022). A new wave of industrialization of PHA biopolyesters. Bioengineering 9, 74. 10.3390/bioengineering9020074 35200427 PMC8869736

[B18] Koller M. Bona R. Chiellini E. Fernandes E. G. Horvat P. Kutschera C. (2008). Polyhydroxyalkanoate production from whey by pseudomonas hydrogenovora. Bioresour. Technology 99, 4854–4863. 10.1016/j.biortech.2007.09.049 18053709

[B19] Koller M. Atlić A. Dias M. Reiterer A. Braunegg G. (2009). “Microbial PHA production from waste raw materials,” in Plastics from Bacteria: Natural Functions and Applications. Springer. 10.1007/978-3-642-03287-5_5

[B20] Kourmentza C. Plácido J. Venetsaneas N. Burniol-Figols A. Varrone C. Gavala H. N. (2017). Recent advances and challenges towards sustainable polyhydroxyalkanoate (PHA) production. Bioengineering 4, 55. 10.3390/bioengineering4020055 28952534 PMC5590474

[B21] Laycock B. Halley P. Pratt S. Werker A. Lant P. (2013). The chemomechanical properties of microbial polyhydroxyalkanoates. Prog. Polymer Science 38, 536–583. 10.1016/j.progpolymsci.2012.06.003

[B22] Lemos P. C. Levantesi C. Serafim L. S. Rossetti S. Reis M. A. Tandoi V. (2008). Microbial characterisation of polyhydroxyalkanoates storing populations selected under different operating conditions using a cell-sorting RT-PCR approach. Appl. Microbiology Biotechnology 78, 351–360. 10.1007/s11356-025-36316-4 18193421

[B23] Lorini L. Martinelli A. Capuani G. Frison N. Reis M. Sommer Ferreira B. (2021). Characterization of polyhydroxyalkanoates produced at pilot scale from different organic wastes. Front. Bioeng. Biotechnol. 9, 628719. 10.3389/fbioe.2021.628719 33681164 PMC7931994

[B24] Magonara C. Montagnese E. Bertasini D. Vona C. Salvatori G. Tayou L. N. (2025). Mixed-culture polyhydroxyalkanoate production with variable hydroxyvalerate content from agri-food residues. Environ. Sci. Pollut. Res. 33, 1–17. 40240659 10.1007/s11356-025-36316-4 PMC12960400

[B25] Mannina G. Presti D. Montiel-Jarillo G. Carrera J. Suárez-Ojeda M. E. (2020). Recovery of polyhydroxyalkanoates (PHAs) from wastewater: a review. Bioresour. Technology 297, 122478. 10.1016/j.biortech.2019.122478 31810735

[B26] Morgan-Sagastume F. Hjort M. Cirne D. Gérardin F. Lacroix S. Gaval G. (2015). Integrated production of polyhydroxyalkanoates (PHAs) with municipal wastewater and sludge treatment at pilot scale. Bioresour. Technology 181, 78–89. 10.1016/j.biortech.2015.01.046 25638407

[B27] Muhammadi SHABINA Afzal M. Hameed S. (2015). Bacterial polyhydroxyalkanoates-eco-friendly next generation plastic: production, biocompatibility, biodegradation, physical properties and applications. Green Chem. Lett. Rev. 8, 56–77. 10.1080/17518253.2015.1109715

[B28] Pagliano G. Galletti P. Samorì C. Zaghini A. Torri C. (2021). Recovery of polyhydroxyalkanoates from single and mixed microbial cultures: a review. Front. Bioeng. Biotechnol. 9, 624021. 10.3389/fbioe.2021.624021 33644018 PMC7902716

[B29] Papa G. Sciarria T. P. Carrara A. Scaglia B. D'Imporzano G. Adani F. (2020). Implementing polyhydroxyalkanoates production to anaerobic digestion of organic fraction of municipal solid waste to diversify products and increase total energy recovery. Bioresour. Technology 318, 124270. 10.1016/j.biortech.2020.124270 33099102

[B30] Pesante G. Magonara C. Zucca A. Montagnese E. Bertasini D. Salvatori G. (2026). Process optimisation for polyhydroxyalkanoates production with tailored hydroxyvalerate content from agri-food residues using mixed microbial cultures. Biotechnol. Biofuels Bioprod. 19, 53. 10.1186/s13068-026-02773-3 42135851 PMC13343652

[B31] Raposo F. Borja R. Cacho J. A. Mumme J. Mohedano Á. F. Battimelli A. (2015). Harmonization of the quantitative determination of volatile fatty acids profile in aqueous matrix samples by direct injection using gas chromatography and high-performance liquid chromatography techniques: multi-laboratory validation study. J. Chromatogr. A 1413, 94–106. 10.1016/j.chroma.2015.08.008 26306912

[B32] Reis M. Serafim L. Lemos P. Ramos A. Aguiar F. VAN Loosdrecht M. (2003). Production of polyhydroxyalkanoates by mixed microbial cultures. Bioprocess Biosystems Engineering 25, 377–385. 10.1007/s00449-003-0322-4 13680343

[B33] Reis M. Albuquerque M. Villano M. Majone M. (2011). Mixed culture processes for polyhydroxyalkanoate production from agro-industrial surplus wastes as feedstocks. Comprehensive Biotechnology. Academic Press, Burlington, Canada; 669.

[B34] Rodrigues A. M. Franca R. D. G. Dionísio M. Sevrin C. Grandfils C. Reis M. A. (2022). Polyhydroxyalkanoates from a mixed microbial culture: extraction optimization and polymer characterization. Polymers 14, 2155. 10.3390/polym14112155 35683828 PMC9182939

[B35] Rodrigues T. Torres C. V. Freitas P. Neves L. A. Carvalheira M. Reis M. A. (2024). Conversion of tomato waste into a poly (3-hydroxybutyrate-co-3-hydroxyvalerate-co-3-hydroxyhexanoate) by a mixed microbial community. J. Environ. Chem. Eng. 12, 114755. 10.1016/j.jece.2024.114755

[B36] Salvatori G. Alfano S. Martinelli A. Gottardo M. Villano M. Ferreira B. S. (2023). Chlorine-free extractions of mixed-culture polyhydroxyalkanoates produced from fermented sewage sludge at pilot scale. Industrial and Eng. Chem. Res. 62, 17400–17407. 10.1021/acs.iecr.3c02684

[B37] Samorì C. Abbondanzi F. Galletti P. Giorgini L. Mazzocchetti L. Torri C. (2015). Extraction of polyhydroxyalkanoates from mixed microbial cultures: impact on polymer quality and recovery. Bioresour. Technology 189, 195–202. 10.1016/j.biortech.2015.03.062 25889806

[B38] Serafim L. S. Lemos P. C. Albuquerque M. G. Reis M. A. (2008). Strategies for PHA production by mixed cultures and renewable waste materials. Appl. Microbiology Biotechnology 81, 615–628. 10.1007/s00253-008-1757-y 19002455

[B39] Siedentop R. Claassen C. Rother D. Lütz S. Rosenthal K. (2021). Getting the most out of enzyme cascades: strategies to optimize in vitro multi-enzymatic reactions. Catalysts 11, 1183. 10.3390/catal11101183

[B40] Sruamsiri D. Thayanukul P. Suwannasilp B. B. (2020). *In* situ identification of polyhydroxyalkanoate (PHA)-accumulating microorganisms in mixed microbial cultures under feast/famine conditions. Sci. Reports 10, 3752. 10.1038/s41598-020-60727-7 32111932 PMC7048717

[B41] Strazzera G. Battista F. Garcia N. H. Frison N. Bolzonella D. (2018). Volatile fatty acids production from food wastes for biorefinery platforms: a review. J. Environmental Management 226, 278–288. 10.1016/j.jenvman.2018.08.039 30121464

[B42] Takahashi S. Tomita J. Nishioka K. Hisada T. Nishijima M. (2014). Development of a prokaryotic universal primer for simultaneous analysis of Bacteria and Archaea using next-generation sequencing. PloS One 9, e105592. 10.1371/journal.pone.0105592 25144201 PMC4140814

[B43] Valentino F. Morgan-Sagastume F. Campanari S. Villano M. Werker A. Majone M. (2017). Carbon recovery from wastewater through bioconversion into biodegradable polymers. New Biotechnology 37, 9–23. 10.1016/j.nbt.2016.05.007 27288751

[B44] Valentino F. Moretto G. Lorini L. Bolzonella D. Pavan P. Majone M. (2019). Pilot-scale polyhydroxyalkanoate production from combined treatment of organic fraction of municipal solid waste and sewage sludge. Industrial and Eng. Chem. Res. 58, 12149–12158. 10.1021/acs.iecr.9b01831

[B45] Van Dyk J. Pletschke B. (2012). A review of lignocellulose bioconversion using enzymatic hydrolysis and synergistic cooperation between enzymes—factors affecting enzymes, conversion and synergy. Biotechnol. Advances 30, 1458–1480. 10.1016/j.biotechadv.2012.03.002 22445788

[B46] Vázquez-Fernández A. Suárez-Ojeda M. E. Carrera J. (2022). Review about bioproduction of Volatile Fatty Acids from wastes and wastewaters: influence of operating conditions and organic composition of the substrate. J. Environ. Chem. Eng. 10, 107917. 10.1016/j.jece.2022.107917

[B47] Villano M. Beccari M. Dionisi D. Lampis S. Miccheli A. Vallini G. (2010). Effect of pH on the production of bacterial polyhydroxyalkanoates by mixed cultures enriched under periodic feeding. Process Biochem. 45, 714–723. 10.1016/j.procbio.2010.01.008

[B48] Volova T. G. Zhila N. O. Kiselev E. G. Sukovatyi A. G. Lukyanenko A. V. Shishatskaya E. I. (2023). Biodegradable polyhydroxyalkanoates with a different set of valerate monomers: chemical structure and physicochemical properties. Int. Journal Molecular Sciences 24, 14082. 10.3390/ijms241814082 37762383 PMC10531092

[B49] Wang X. Oehmen A. Freitas E. B. Carvalho G. Reis M. A. (2017). The link of feast-phase dissolved oxygen (DO) with substrate competition and microbial selection in PHA production. Water Res. 112, 269–278. 10.1016/j.watres.2017.01.064 28183066

[B50] Wang Z. Xu H. Mei Y. Suo M. Hrynsphan D. Tatsiana S. (2026). Chain-elongation routes to caproic acid toward industry-ready continuous operation and low-energy recovery. Bioresour. Technol. 444, 133974. 10.1016/j.biortech.2026.133974 41513174

[B51] Yang J.-K. Ding M.-Y. Hu Y.-T. Hong X.-Z. Han Z.-G. Lei L. (2025). Genome-wide analysis of Flavobacterium strain YJ01 demonstrates abundant enzymes synergistically degrade diverse nature carbohydrates. J. Appl. Microbiol. 136, lxae309. 10.1093/jambio/lxae309 39749830

[B52] Zhou M. Yan B. Wong J. W. Zhang Y. (2018). Enhanced volatile fatty acids production from anaerobic fermentation of food waste: a mini-review focusing on acidogenic metabolic pathways. Bioresour. Technology 248, 68–78. 10.1016/j.biortech.2017.06.121 28693950

[B53] Zou Y. Yang M. Tao Q. Zhu K. Liu X. Wan C. (2023). Recovery of polyhydroxyalkanoates (PHAs) polymers from a mixed microbial culture through combined ultrasonic disruption and alkaline digestion. J. Environ. Manag. 326, 116786. 10.1016/j.jenvman.2022.116786 36410150

